# HIV controllers: hope for a functional cure

**DOI:** 10.3389/fimmu.2025.1540932

**Published:** 2025-02-25

**Authors:** Zhuoya Deng, Hongxia Yan, Olivier Lambotte, Christiane Moog, Bin Su

**Affiliations:** ^1^ Beijing Key Laboratory for HIV/AIDS Research, Clinical and Research Center for Infectious Diseases, Beijing Youan Hospital, Capital Medical University, Beijing, China; ^2^ Sino-French Joint Laboratory for HIV/AIDS Research, Sino-French Joint Laboratory for Research on Humoral Immune Response to HIV Infection, Beijing Youan Hospital, Capital Medical University, Beijing, China; ^3^ University Paris Saclay, AP-HP, Bicêtre Hospital, UMR1184 INSERM CEA, Le Kremlin Bicêtre, France; ^4^ Laboratoire d’ImmunoRhumatologie Moléculaire, Institut National de la Santé et de la Recherche Médicale (INSERM) UMR_S 1109, Institut Thématique Interdisciplinaire (ITI) de Médecine de Précision de Strasbourg, Transplantex NG, Faculté de Médecine, Fédération Hospitalo-Universitaire OMICARE, Fédération de Médecine Translationnelle de Strasbourg (FMTS), Université de Strasbourg, Strasbourg, France; ^5^ Central Laboratory of Beijing Youan Hospital, Capital Medical University, Beijing, China

**Keywords:** HIV controllers, functional cure, immune response, genetic polymorphisms, HIV reservoirs

## Abstract

Elite controllers (ECs) and post-treatment controllers (PTCs) represent important models for achieving a functional cure for HIV. This review synthesizes findings from immunological, genetic, and virological studies to compare the mechanisms underlying HIV suppression in ECs and PTCs. Although ECs maintain viral control without antiretroviral therapy (ART), PTCs achieve suppression following ART discontinuation. Both groups rely on adaptive and innate immunity, host genetic factors, and characteristics of the HIV reservoir; however, they exhibit distinct immune responses and genetic profiles. These differences provide insights into strategies for sustained ART-free remission. Understanding the shared and unique mechanisms in ECs and PTCs can inform the development of novel therapeutic approaches, including immune-based therapies and genome editing, to achieve a functional cure for HIV-1.

## 1 Introduction

Current antiretroviral therapy (ART) effectively suppresses plasma viral RNA to undetectable levels for extended durations, preventing viral evolution. ART is generally prescribed for interventions during the chronic phase of infection. However, in most patients, a rapid rebound in viremia is observed within weeks of ART interruption (1, 2). This dependence poses several challenges, including side effects, drug resistance, stigma, and economic burden. Consequently, researchers are exploring various therapeutic strategies to prevent or delay viral rebound following treatment interruption, aiming for post-treatment remission or a functional cure for HIV (3). A functional cure for HIV refers to a state where patients can cease ART. The main research directions include gene therapy and immunotherapy. These treatment methods aim to fundamentally change the body’s ability to control HIV to achieve long-term virus control without ART (4). Despite substantial progress, achieving sustained ART-free remission remains elusive for the majority of HIV-infected individuals.

Special populations such as elite controllers (ECs) and post-treatment controllers (PTCs) offer invaluable insights into the mechanisms of ART-free HIV remission. ECs, who are able to maintain viral suppression (<50 copies/mL) without initiating ART for prolonged periods (5), provide potential directions for a functional cure (6). The existence of HIV ECs indicates that the goal of ART-free HIV remission is possible, likely because of favorable genetic and immunological profiles (7). However, they represent a very small subset of the population (<1%) (8–10). In contrast, PTCs, identified in therapeutic intervention studies, sustain low or undetectable viremia following ART discontinuation and constitute a larger proportion of patients (5–15% in some studies) (11, 12). The existence of ECs and PTCs demonstrates the feasibility of ART-free remission and inspires efforts to emulate these natural mechanisms of control in broader patient populations.

Although both ECs and PTCs achieve viral suppression without continuous ART, the mechanisms underlying their control may differ. ECs often rely on robust HIV-specific immune responses (13, 14), advantageous host genetic factors (15), and attenuated viral (16) characteristics. On the other hand, PTCs appear to achieve control through a combination of acute ART initiation, reduced HIV reservoir size, and immune-mediated mechanisms (11, 17, 18). Comparing and contrasting these groups provides critical insights into diverse pathways for achieving functional HIV cures.

This review aims to elucidate the overlapping and distinct mechanisms of HIV suppression in ECs and PTCs, with a focus on identifying therapeutic targets and strategies for achieving ART-free remission. By examining these two populations, we hope to provide guidance for future therapeutic approaches, including immune-based therapies and genome-editing strategies. Ultimately, the aim of this study is to contribute to the ongoing effort to achieve a functional cure for HIV-1.

## 2 Host−virus interaction

**Figure 1:**
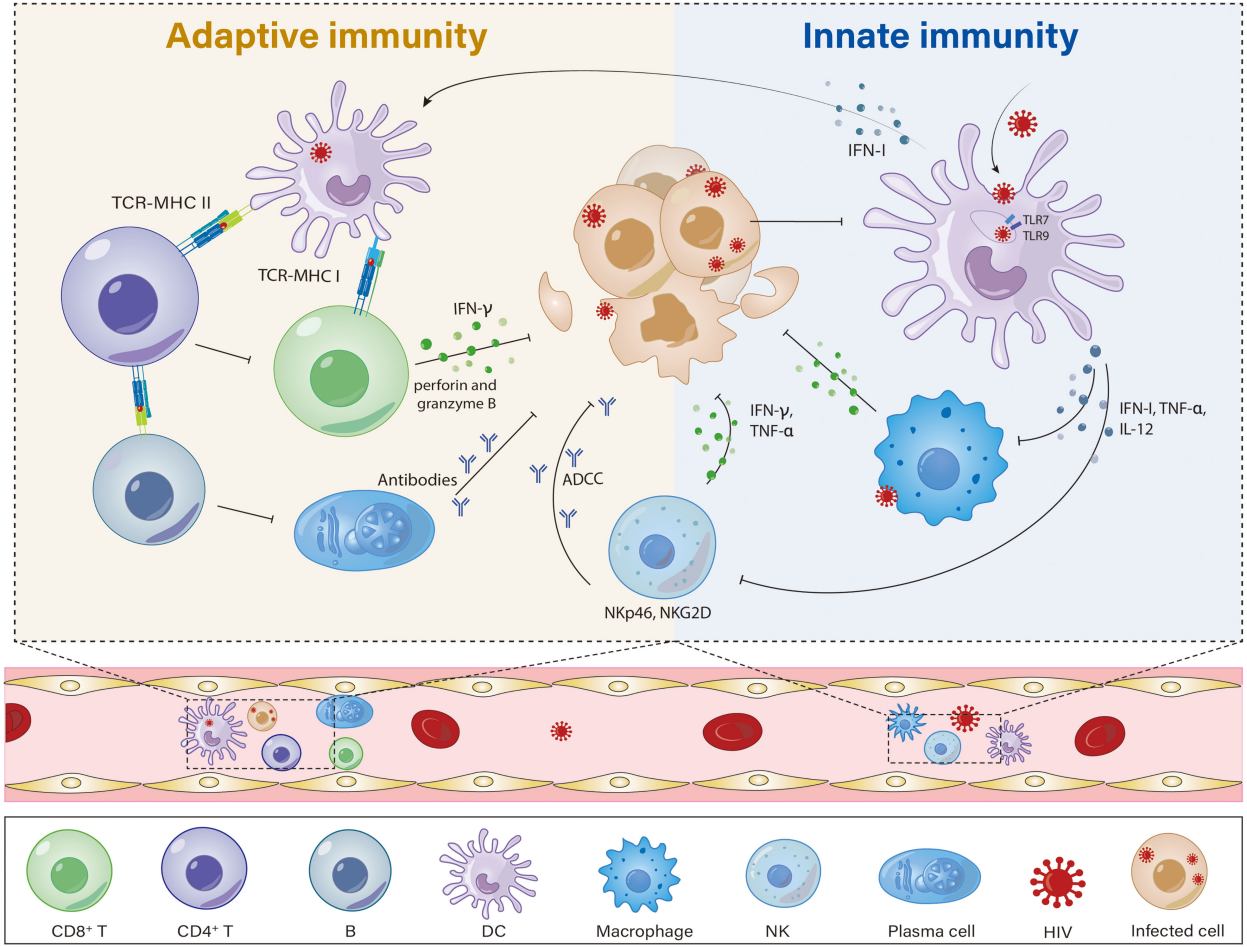
Potential mechanisms of innate immunity and adaptive immunity possibly contributing to the spontaneous control of viremia in HIV controllers. HIV controllers have a more robust and effective immune response, with functional DCs and NKs, highly cytotoxic and activated CD8^+^ T cells, and CD4^+^ T cells that can better maintain immune function. (1) DCs from HIV controllers are less permissive to HIV-1 infection but more efficient at capturing HIV-1 particles, and they have distinct phenotypes and functions, such as the presence of a highly functional CD64^hi^ PD-L1^hi^ mDC state, enhanced antigen presentation, and improved sensing of cytosolic HIV-1 replication products, which can trigger stronger T-cell responses. (2) CD4^+^ T cells, which are the main target of HIV, are severely depleted in AIDS patients. In HIV controllers, CD4^+^ T cells maintain their numbers and functions, are less sensitive to HIV-1 infection, and have high - affinity TCRs. (3) In HIV controllers, CD8^+^ T cells show stronger HIV antigen - induced cell proliferation, cytotoxic activity, and can effectively inhibit HIV-1 replication in autologous CD4^+^ T cells. (4) In HIV controllers, NK cell activity is enhanced. These NK cells secrete higher levels of IFN-γ and TNF-α, express more NKp46/NKG2D-activated receptors, and possess a stronger ADCC capacity.

In the early stages of HIV infection, immune activation plays a crucial role in controlling HIV replication and may contribute to limiting the establishment of viral reservoirs (**Figure 1**). This activation primarily results from the immune system’s response to the presence of the virus. However, prolonged and sustained immune activation, especially in the chronic phase of HIV infection, can impair immune reconstitution, accelerate immune senescence and increase the risk of non-AIDS-related diseases (19). Understanding these complex immune mechanisms is critical, particularly in ECs and PTCs, whose immune responses provide insights into strategies for improving clinical outcomes. This section focuses on the immune characteristics of ECs and PTCs, explores how adaptive immune mechanisms, particularly cellular immunity, contribute to the control of HIV infection and offer potential avenues for immunotherapy (**Table 1**).

**Table 1:** Immunity comparison Items between ECs and PTCs.

Comparison Items	ECs	PTCs
Formation Mechanism	Naturally control the virus without any ART (5).	Viral control was achieved after ART (11, 12).
Viral Load	The viral load is very low or undetectable and relatively stable (5).	Treatment may remain low or even undetectable for a long time, but there is a risk of rebound (11, 12).
CD8^+^ T cell	With potent antiviral activity and can effectively control viral replication (20).	Antiviral activity was relatively weak but still maintained viral suppression after treatment (11, 21).
CD4^+^ T cell	Counts are usually near normal levels and function relatively normal to provide immunological support (22, 23).	PTCs exhibit reduced activation of mature CD4^+^ T cells but more robust Gag-specific CD4^+^ T-cell activity (18).
Humoral immunity	ECs may produce stronger neutralizing antibody responses. Antibodies from ECs may also be involved in other immune mechanisms, such as ADCC, which remove virus-infected cells by the activation of immune cells (24–26).	The neutralizing antibody response was relatively weak, but it may also contribute to viral suppression (27, 28).
NK cell	Activity and function may be stronger (29).	NK cells derived from PTCs secreted higher levels of IFN-γ and showed a strong ability to control HIV infection (30, 31).
DC	DCs are relatively mature and more functional enough to efficiently uptake, process, and present antigens and activate T cells and other immune cells (32, 33).	The feature is relatively unclear.
Dependence on treatment	There is generally no need for treatment (5).	Long-term ART is required for the maintenance status (11, 12).

### 2.1 Adaptive immunity

#### 2.1.1 Cellular immunity

##### 2.1.1.1 CD8^+^ T cells

By exhibiting potent virus-specific responses, CD8**^+^** T cells play a crucial role in the immune surveillance of HIV, especially in ECs (6), by interacting synergistically with HLA genes, restricting viral mutations, and performing versatile immune functions. HIV-1-specific CD8^+^ T cells are widely regarded as the key mediators of long-term viral suppression in HIV-1 ECs (20). These T lymphocytes recognize and eliminate cells expressing non-self-peptides using major histocompatibility complex (MHC) molecules, which are also referred to as HLAs. Genome-wide association studies (GWASs) have identified specific protective HLA class I alleles, particularly HLA-B* 57/58 and HLA-B* 27, that are strongly associated with CD8^+^ T-cell responses (7, 34). Consistent with many early cohort studies, the majority of ECs possess these protective HLA alleles (35). Notably, the function of HIV-specific CD8^+^ T cells in ECs is qualitatively distinct from that of chronic HIV progressors. One major difference is the upregulation of both inflammatory chemokine gene expression and effector functions in ECs. These functional adaptations result in enhanced HIV antigen-induced cell proliferation and robust cytotoxic activity, which collectively contribute to effective viral suppression in ECs (36). In addition to their cytolytic functions, these CD8^+^ T cells secrete a variety of cytokines and undergo activated degranulation upon HIV peptide presentation (37). For example, CXCR5^+^ follicular CD8^+^ T cells release perforin and granzyme B near HIV RNA^+^ cells in lymphoid follicles (38). Crucially, the HLAs of HIV-specific CD8^+^ T cells in ECs facilitate the efficient presentation of conserved and highly networked HIV-1 epitopes, thereby enhancing T-cell-mediated immune responses. These findings highlight the central role of HLA class I-restricted CD8^+^ T-cell responses in the control of HIV-1 replication in ECs. Furthermore, one of the primary functional differences between ECs and HIV progressors lies in the ability of CD8^+^ T cells in ECs to effectively inhibit HIV-1 replication in autologous CD4^+^ T cells (39, 40), even when they are infected *in vitro* (41).

Moreover, other studies suggest that the increased loading of lytic granules within CD8^+^ T cells in ECs results in a substantial increase in the delivery of granzyme B, which is directed at HIV-infected cells (36, 42, 43). In addition to these cytolytic functions, CD8^+^ T cells in ECs exhibit HIV-1-specific activation, which is associated with distinct metabolic (44) and transcriptional profiles (45). These profiles include the differential expression or regulation of key transcription factors, further highlighting the complexity of CD8^+^ T-cell responses in ECs. Additionally, viruses in EC plasma exhibit different mutations compared with those found in resting CD4^+^ T cells, indicating that CD8^+^ T cells exert active immune selection pressure on the virus (46–48).

PTCs may exhibit distinct immunological characteristics in contrast to ECs, with features such as attenuated cellular and humoral antiviral responses and increased heterogeneity (21). Compared with ECs, HIV-specific CD8^+^ T cells in PTCs presented decreased activation levels, lower frequencies, and a reduced ability to inhibit the infection of autologous CD4^+^ T cells (11). Moreover, research has revealed that PTCs are heterogeneous and that effective HIV-specific CD8^+^ T-cell-mediated responses can be detected in these patients (49). A previous study revealed that rhesus macaques achieved potential PTC effects after treatment. Rhesus macaques (*Macaca mulatta*) achieving PTC effects exhibited lower viral DNA levels in deep lymph nodes (e.g., mesenteric and parenteral lymph nodes), correlating with stable virologic suppression. In contrast, rhesus macaques with higher viral DNA levels in superficial lymph nodes (e.g., cervical or axillary lymph nodes) showed stable or unstable viral rebound. Tissues from PTC rhesus macaques demonstrated significantly reduced quantitative viral outgrowth, fewer Programmed Death-1 (PD-1)^+^ central memory CD4^+^ T cells, and CD8^+^ T cells critically contributed to maintaining virologic control efficacy (50).

##### 2.1.1.2 CD4^+^ T cells

CD4^+^ T cells have specific characteristics in controllers. CD4^+^ T helper cells are required for long-term maintenance of antigen-specific CD8^+^ T cells. CD4^+^ T cells play an important role in HIV infection. They are the primary target cells of HIV but also recognize viral antigens and activate other immune cells, such as CD8^+^ T cells and B cells. In the early stages of infection, the number of CD4^+^ T cells decreases sharply, resulting in immune system damage. However, in ECs, the number and functions of CD4^+^ T cells are maintained, which is related to the unique characteristics of CD4^+^ T cells. CD4^+^ T cells have high-affinity T-cell antigen receptors (TCRs) that effectively recognize high-avidity T cells and respond to low amounts of HIV viral antigens (22). In addition, the levels of certain cytokines, such as Chemokine (C-C motif) ligand (CCL) 14, CCL21, CCL27, Chemokine (C-X-C motif) ligand (CXCL) 1 and CXCL12, are elevated in ECs, and these cytokines upregulate cell activation, HIV coreceptor expression and the effector functions of CD4^+^ T cells (51).

Compared with those in HIV-1 progressors and HIV-1-negative individuals, CD4^+^ T cells from ECs are less sensitive to HIV-1 infection. The intrinsic mechanisms that limit HIV-1 replication in CD4^+^ T target cells may also play an important role in mediating the resistance of ECs to HIV-1 infection. In the process of HIV infection, CD4^+^ T-cell number and function are important indicators for assessing disease progression and for determining whether a patient is a controller. ECs (plasma viral load low to undetectable, median percentage of CD4^+^ T cells> 40%) have normal T-cell and monocyte phenotypes and therefore may have limited benefit from ART (23).

In addition to maintaining CD4^+^ T-cell functions, ECs also present an increased frequency of helper T (Th) 17 cells and activated regulatory T (Treg) cells compared with ART-treated and HIV-1-negative individuals (52). These subsets are more readily activated and produce higher levels of cytokines, further bolstering the immune response in ECs. Notably, ECs maintain a high Th17/Treg ratio, which has been shown to be beneficial for controlling inflammation and sustaining immune balance (52, 53). Research indicates that HIV-1-specific CD4^+^ T cells in ECs robustly express genes associated with Th1, Th17, and Th22 subsets of helper T cells, suggesting a multifaceted immune response. Moreover, the expression of cytokines related to mucosal immunity is increased in the HIV-specific CD4^+^ T cells of ECs, in contrast with the profiles observed in chronic progressors (54).

PTCs exhibit reduced activation of mature CD4^+^ T cells but more robust Gag-specific CD4^+^ T-cell activity (18). Compared with noncontrollers (NCs), PTCs subjected to early analytical treatment exhibited significantly elevated levels of Gag-specific CD4^+^ T cells that produce Interferon-γ (IFN-γ), along with a modest increase in CD4^+^ T cells that produce Interleukin-2 (IL-2). In addition, CD4^+^ T cells also have important implications for HIV vaccine research, as they are the main targets of vaccine-induced immune responses (**Figure 1**).

#### 2.1.2 Humoral immunity

An increasing number of studies support a role for humoral immunity in controlling HIV infection and replication (55, 56). In particular, ECs exhibit efficient humoral responses that contribute significantly to the natural control of HIV-1, with notably high levels of HIV-specific memory B cells (57). These responses are crucial for long-term viral suppression.

One of the key characteristics of ECs is their production of neutralizing antibodies (NAbs). Although the level of NAbs in ECs may be lower than that in chronic progressors (24, 25), their higher affinity and polyfunctional activity may still allow them to exert strong neutralizing activity. This finding suggests that strong suppression of viral replication limits stimulation and that the maintenance of effective NAb responses and high-titer NAbs are not required for maintenance of viral suppression (58). Although NAbs might not be the primary factor controlling viral replication, they may still play a role in the natural control of HIV-1. For example, bNAbs in ECs are capable of neutralizing multiple strains of HIV, including diverse subtypes, by targeting conserved regions of the virus, such as the CD4 binding site, V3 loop, and gp120/gp41 interface (59).

In addition to bNAbs, ECs present increased levels of nonneutralizing antibodies that engage in Fc-mediated effector functions such as antibody-dependent cellular cytotoxicity (ADCC). In these individuals, ADCC levels correlate positively with CD8^+^ T-cell function, suggesting that effective CD4^+^ T-cell support and that CD8-mediated suppression enhance ADCC activity (60). Moreover, these antibodies are thought to contribute to more effective control of viral replication in ECs than chronic progressors (26). For example, negative factor (Nef) proteins in ECs have a diminished ability to downregulate CD4, enhancing the exposure of ADCC-mediated epitopes on the HIV-1 envelope (Env) and increasing the susceptibility of HIV-infected cells to ADCC clearance (61), providing a potential therapeutic strategy.

Memory B-cell responses in ECs also play a critical role in controlling HIV. Research by Rouers et al. revealed that ECs maintain robust memory B-cell compartments and HIV-specific memory B-cell responses (62). The frequency of Env-specific B cells in HLA-B*57^+^ ECs correlates with the breadth of neutralization observed, suggesting that these memory responses are crucial for maintaining broad neutralization capacities and the natural control of HIV infection.

Some studies have shown that in PTCs, the humoral immune profiles are heterogeneous, mainly affected by virus exposure and dynamics (63). Virally-exposed PTCs produce a functionally coordinated and effective humoral response to HIV-1, which has the potential to generate bNAbs and autologous NAbs. For example, Molinos-Albert et al. identified a bNAb lineage, EPTC112, in a PTC with an elite neutralizer profile, underscoring the potential role of antibodies in long-term HIV control (27, 28). These antibodies demonstrated the ability to neutralize the autologous virus even in the presence of escape mutations, indicating a complex immune response that may support functional remission strategies after ART interruption.

In summary, although NAb levels in ECs may be lower than those in chronic progressors, their higher affinity and greater ADCC activity likely contribute to more effective control of HIV-1. Although bNAbs may not be the decisive factor in regulating viral replication, they may still play an important role in the natural control of HIV infection, especially in individuals not receiving ART. ECs exhibit a multifaceted humoral immune response characterized by strong neutralizing antibody activity, enhanced memory B-cell responses, and potentially structural modifications to the virus that aid in controlling HIV-1 infection without ART. These findings underscore the importance of humoral immunity in HIV-1 control; however, more research is needed to fully understand its role across different patient populations.

### 2.2 Innate immunity

Research indicates that HIV-1 immune control is primarily mediated by virus-specific T-cell responses, where increased T-cell polyfunctionality is associated with improved viral control during HIV-1 disease progression. However, innate immune cells are also involved in the natural control of HIV-1 (**Figure 1**). Innate immunity serves as the first line of defense against HIV and triggers subsequent adaptive immune responses. Pattern recognition receptors recognize HIV, and a series of immune cells are subsequently recruited to induce or activate numerous innate immune-related factors to exert antiviral effects. In HIV-infected EC/PTC populations, dendritic cells (DCs), natural killer (NK) cells, macrophages, and NKT cells are crucial components of innate immunity and play important roles in controlling HIV. A discussion of innate immunity in HIV controllers is provided in the following sections.

#### 2.2.1 Natural killer cells

Accumulating evidence indicates that NK cells play an important role in HIV control. First, increased NK cell frequency and activity were observed in HIV controllers than in NCs (29). In ECs, NK cells exhibit several distinct characteristics distinguishing them from NCs. Pohlmeyer et al. used mass cytometry to analyze NK cell phenotypes in ECs and viremic non-controllers (VNCs). They revealed that the CD11b^+^CD57^-^CD161^+^Siglec-7^+^ subpopulations of CD56^dim^CD16^+^ NK cells were more abundant in ECs and HIV-negative controls than in VNCs and that the frequency of these cells correlated with HIV DNA levels (64). Certain alleles of killer immunoglobulin receptors (KIRs), such as KIR3DS1, on NK cells are associated with the slow disease progression (65, 66). Marras et al. investigated the effects of NK cell functional characteristics on controllers [ECs and long-term nonprogressors (LTNPs)] and progressor patients. HIV DNA copy numbers (either total or integrated) in circulating CD4^+^ T cells were negatively correlated with transcriptionally unique NK cell functions. Specifically, the production of induced IFN-γ and the expression of NKp46/NKp30-activating receptors are inversely associated with HIV reservoir size (67). Moreover, in an African green monkey model of nonpathogenic SIV infection, NK cells migrate into follicles and play a major role in controlling the HIV reservoir in lymph nodes (68). These results suggest that some specific phenotypes and functions of NK cells contribute to the control of HIV reservoirs in ECs.

Recent studies of the VISCONTI cohort revealed high levels of specific NK cells in PTCs. In functional studies *in vitro*, NK cells derived from PTCs also secreted relatively high levels of IFN-γ and showed a strong ability to control HIV infection (30). A proviral landscape study of PTCs revealed higher NK cell activation levels in PTCs, which was associated with lower levels of total and defective proviral genomes (31). Additionally, in an analytical treatment interruption (ATI) study of PTCs from AIDS Clinical Trials Group (ACTG), immunologically, PTCs exhibited stronger NK cell activation and function. PTCs had increased levels of activation markers, including CD38^+^ CD56^+^ NK cells and CD69^+^ NK cells (18). Some studies involving NK cell analysis in controllers revealed that the CD56^+^/CD16^-^ NK cell subsets of controllers were the same as those of healthy donors and greater than those of chronic patients and that the IFN-γ, Tumor Necrosis Factor-α (TNF-α) and IL-12 levels secreted from the NK cells of controllers were increased (69, 70). Moreover, HIV controllers expressed higher Natural Killer Group 2, Member D (NKG2D) levels than chronic patients, enhancing the susceptibility of infected cells to ADCC (69, 71). Recent studies have treated chronic HIV infection with NK cells coupled with cytokines, indicating the potential of NK cells in controlling HIV (72). These results suggest that preserving the phenotype and function of NK cells at the time of treatment is important for HIV control. Transcriptionally and functionally distinctive NK cell characterization can be used to prospectively identify HIV-infected patients who are highly likely to successfully receive ART simplified to monotherapy or ART interruption (PTCs).

#### 2.2.2 Dendritic cells

The majority of HIV-1 ECs restrict virus replication by eliciting robust HIV-1-specific T-cell responses, and DCs stand out as the most potent natural antigen-presenting cells, playing a pivotal role in the induction and maintenance of antigen-specific T-cell responses (32, 33). Over the past few years, the role of DCs in HIV-1 controllers has been increasingly appreciated. The DCs derived from HIV controllers are less permissive to HIV-1 infection than cells obtained from healthy donors or HIV-1 patients after ART treatment, but DCs from HIV controllers have a strong ability to capture HIV-1 particles (73). Phenotypically, a highly functional CD64^hi^PD-L1^hi^ mDC state was found in ECs using single-cell sequencing, with the fractional abundance associated with increased CD4^+^ T-cell counts and a decreased HIV-1 viral load, and it effectively triggered polyfunctional T-cell responses *in vitro* (74). Mass cytometry analysis revealed that plasma HIV RNA levels were positively associated with a loss of mDC and pDC subpopulations that displayed high expression of leukocyte immunoglobulin-like receptors (LILRs). A particular subtype of CD1c^+^ CD32b^hi^ HLA-DR^hi^ mDCs in peripheral blood monouclear cells (PBMCs) was enriched in HIV ECs (75). mDCs from ECs have significantly enhanced antigen presentation characteristics and are able to sensitize allologous T cells more efficiently than those from healthy donors or HIV-1 progressors (76). Cytosolic immunorecognition of HIV-1 in mDCs promotes the initiation and expansion of HIV-1-specific T cells. HIV-1 prevents intracellular immune recognition by mDCs in most infected individuals. However, mDCs from ECs exhibit an improved ability to sense cytosolic HIV-1 replication products. In ECs, HIV-1 replication products in mDCs result in rapid and sustained secretion of endogenous IFN-I from mDCs and the induction of potent HIV-1-specific CD8^+^ T cells (77, 78). This finding suggests that endogenous cellular IFN-I secretion in DCs plays an important role in inducing potent HIV-1-specific CD8^+^ T cells and may contribute to eliciting functional T-cell immunity against HIV-1 for prophylactic or therapeutic clinical purposes. The sterile alpha motif and HD domain-containing protein 1 (SAMHD1) restricts HIV-1 infection of mDCs and other myeloid cells (79). SAMHD1 is a host protein that is highly expressed in myeloid cells and limits HIV-1 replication at the reverse transcription level (80) by depleting the intracellular pool of deoxynucleoside triphosphates (81) and directly degrading viral RNA (82). In addition, intracellular immune recognition of HIV-1 by mDCs in ECs involves detection of the replication products of viral RNA and DNA. Therefore, the DNA sensor cyclic GMP-AMP synthase (cGAS) (83), the RNA sensor Retinoic acid-inducible gene I (RIG-I) (84) and cooperation between the two sensing pathways can improve the innate recognition of HIV-1 by mDCs in ECs (85). Despite lower Ag uptake than that of mDC subsets, pDCs efficiently cross-present exogenous Ags to CD8^+^ T cells (86). pDCs are bone marrow-derived cells that sense HIV *via* Toll-like receptor (TLR)-7 and TLR-9 and convert this signal to IFN-I (IFN-α, -β, -ϵ, -ω and -κ) production and T-cell activation (87, 88). Compared with VNCs, pDCs from ECs have a greater capacity to reduce HIV production and induce T-cell apoptosis, whereas pDCs from viremic NCs minimally respond to previous TLR-9 stimuli. Additionally, the function of pDCs from ECs is preserved, with similar levels of IFN-α production in healthy donors and higher levels compared to those in VNCs (89, 90). Together, these findings suggest a specific link between innate and adaptive HIV-1 immunity in controllers and that DCs may play an important role in immune defense mechanisms and HIV-1 therapy.

Increasing evidence suggests that immune-mediated effector responses play a crucial role in the establishment of deep viral latency, a state in which the virus remains dormant within cells. The study of immune-mediated effector responses in HIV controllers offers promising insights into the complex interactions between the immune system and HIV-1. HIV controllers are characterized by robust immune responses mediated by a combination of both innate and adaptive immunity and are able to effectively target and eliminate HIV-1-infected cells. The innate immune system is able to quickly recognize and respond to HIV-1, whereas the adaptive immune system generates specific T-cell responses that target and destroy infected cells (**Figure 1**). As we continue to unravel the immune mechanism of this remarkable subgroup, we hope to identify new approaches to harness the power of the immune system to combat HIV-1.

## 3 Genetic variation

Susceptibility to HIV infection and the rate of disease progression vary significantly among individuals. After initial infection, the HIV RNA levels reached and the subsequent progression to AIDS differ widely. A growing number of studies suggest that genetic differences may contribute to this variability, influencing not only susceptibility to HIV but also the rate of progression to disease. Additionally, genetic factors may be involved in the risk of developing specific HIV-related complications, such as renal or neurological disorders, as well as non-AIDS conditions such as cardiovascular disease. Furthermore, certain genetic variants have been linked to the recovery of CD4^+^ T lymphocyte counts following ART. Through candidate gene approaches and GWASs, several key genetic variations associated with both HIV susceptibility and progression have been identified (**Table 2**). These findings provide valuable insights into HIV control.

**Table 2:** Potential host genetic factors involved in viral suppression in HIV controllers.

Gene	Mechanism	References
HLA-B*57/58 and HLA-B*27	Presentation of specific HIV antigens; lower viral load	(13, 91)
*HLA-Bw4* and *KIR*	HLA-Bw4 provides a ligand to the activated KIR. The host KIR genotype determines the HIV-mediated changes in the NK cell repertoire. KIR3DL1CD8^+^ T cells with strong early activation and proliferation may, together with KIR3DL1CD69^+^ NK cells, play a protective role during acute/early HIV infection in individuals homozygous for Bw4.	(92–94)
HLA-DRB1*15:02	Reduced response of CD4^+^ T cells to HIV Gag and Nef proteins; lower viral load.	(95)
*HLA-C*	Individuals carrying the HLA-C rs9264942 CC genotype (SNP 35 kb upstream of HLA-C) showed a significantly decreased HIV-1 viral load. Decreased viral load set point.	(96–98)
*MICA*	Affects the presentation of the HLA I peptides; linkage to the protective HLA-B allele; a noncoding SNP (rs4418214) near MICA is enriched in HIV-1 controllers.	(99–101)
*PSORS1C3*	The rs3131018 SNP in PSORS1C3 is a Genetic determinant of HIV-1 control that affects the presentation of HLA I peptides.	(7, 102)
*HCP5*	Linkage disequilibrium with HLA-B*57:01; lower HIV viral load.	(103–106)
*ZNRD1*	Interference in the processing of HIV transcripts; influences ZNRD1 expression; linkage disequilibrium with HLA-A10.	(106–108)
*ZNF*	Viral integration sites are more frequently present near ZNF genes on chromosome 19, which are often marked by tightly packed repressive chromatin, associated with suppressed viral replication in ECs.	(109–111)
*CCR5*	Δ32 allele deletion/lower CCR5 expression; reduction in virus entry into the cells.	(112–114)
*CXCR6*	CXCR6 is downregulated in ECs; low prevalence of rs2234358-T in LTNPs; trafficking of effector T cells and activation of NK T cells.	(115–118)
*TRIM5*	The rs10838525 SNP in TRIM5α may contribute to viral suppression among HIV-1 ECs; control of the chronic viral infection in HIV-1 controllers is mediated by the autophagy mechanism; it defends against invading HIV-1.	(119, 120)
*APOBEC3G/3F*	Destructive cytosine to uracil changes catalyzed by APOBEC3G/3F during reverse transcription of HIV-1 RNA into DNA; reduced innate immune restriction of HIV-1 replication.	(110, 121)

### 3.1 CCR5

C-C chemokine receptor type 5 (CCR5) is a critical coreceptor used by HIV-1 to enter immune cells. Given its key role in the viral entry process, targeting CCR5 has emerged as a promising strategy for preventing and potentially curing HIV-1 infection. Targeting the CCR5 receptor to decrease host cell susceptibility or confer resistance to infection could enhance HIV-1 inhibition, especially when used alongside other anti-HIV-1 strategies (122).

One of the most notable genetic findings in HIV research is the CCR5 Δ32 mutation, which involves a 32-base pair deletion in the CCR5 gene. This mutation leads to a truncated protein that is not expressed on the cell surface, rendering individuals homozygous for the Δ32 allele resistant to HIV-1 infection. These findings were highlighted in the cases of the “Berlin” and “London” patients, where stem cell transplants from CCR5 Δ32 homozygous donors resulted in long-term HIV remission. This has driven interest in exploring CCR5-targeted therapies for broader HIV-1 treatment options (123–125). Recent studies have shown that the downregulation of CCR5 expression in HIV-specific CD4^+^ T cells leads to the natural ability of ECs to control HIV-1 replication (112–114). This reduced expression limits the ability of the virus to enter and infect these cells, suggesting that inactivating or downregulating the CCR5 gene in a nonfunctional receptor could be key to achieving a functional HIV cure. Recent studies have demonstrated that adoptive cellular therapy using CCR5 knockout in autologous T cells can achieve sustained HIV control in some patients (126, 127).

### 3.2 HLA

Human leukocyte antigen (HLA) class I molecules play important roles in the immune response against HIV, particularly in ECs and PTCs. Genetic variants in HLA-B are strongly correlated with both favorable and adverse outcomes in HIV infection patients. Specifically, certain HLA-B alleles, such as HLA-B*57/58 and HLA-B*27, are associated with favorable immune control, whereas others, such as HLA-B*35, are linked to faster disease progression and higher viral loads (7, 34, 91).

HLA-B*57 and HLA-B*27 are considered protective alleles in HIV infection. These alleles are found more frequently in individuals who are classified as LTNPs and ECs. In ECs, specific HLA class I haplotypes, particularly HLA-B*57-01 and HLA-B*27-05, are notably overrepresented. The presence of these alleles is believed to contribute to the lower viral replication observed in these individuals. This control may be partially attributed to the induction of a robust cell-mediated immune response against HIV, including CD8^+^ T-cell responses that effectively target HIV epitopes (91).

HLAs are associated with the degree of immune control in HIV-infected patients, especially in elite controls. However, unlike those associated with HIV ECs, the proportion of favorable HLA alleles associated with viral control is not high in PTCs (31). In contrast, a number of PTCs have the HLA-B*35 allele, which is associated with faster progression to AIDS and a greater viral load in general. HLA-B35 haplotype enrichment in PTCs might be explained by its association with other genetic signatures. Moreover, not all HLA-B*57^+^ or HLA-B*27^+^ patients become HIV controllers when infected. In different cohorts, a significant proportion of HIV controllers do not carry these protective HLA alleles. For example, 15% to 70% of controllers in some cohorts are not HLA-B*57^+^ (128). This finding indicates that other factors also contribute to the ability to control HIV replication.

Even among HLA-B*57^+^ controllers, heterogeneity in the ability of CD8^+^ T cells to suppress viral replication is observed. Some HLA-B57^+^ individuals are strong viral suppressors, whereas others are weak suppressors. The frequency of HIV-specific CD8^+^ T cells is highly dependent on the viral burden in HLA-B*57^+^ patients (128). In those with a lower viral load (weak suppressors), the frequencies of these cells are much lower, suggesting that the level of antigen stimulation also plays a role in the magnitude of the immune response.

## 4 Unusual HIV reservoir

One of the most notable features of both ECs and PTCs is the size and composition of their HIV reservoirs. Understanding these reservoirs is crucial for advancing HIV cure strategies given that the size and integrity of the viral reservoirs directly influence the potential for achieving sustained remission without ART (129). ECs are characterized by a very small HIV reservoir, particularly among CD4^+^ T cells. This is a common feature observed across natural ECs and PTCs, which underscores the importance of reducing the HIV reservoir size in patients undergoing ART as part of HIV cure efforts.

The activation and clearance of HIV-1 viral reservoirs is an important strategy for the functional cure of AIDS (130, 131). In ECs, HIV integrates into the host genome, but the quantity and genetic integrity of the proviruses in their CD4^+^ T cells are significantly lower than those in chronic progressors and ART-treated patients (132, 133). Studies have shown that proviral reservoirs of ECs usually consist of oligoclonal to near monoclonal clusters of intact proviral sequences (109). These findings suggest that under immune pressure, ECs may favor the persistence of smaller, less inducible viral reservoirs, avoiding the transcription of intact proviruses. This selective process may help limit viral replication and prevent the activation of viral reservoirs, contributing to the spontaneous control of HIV without ART (134).

In ECs, intact proviruses tend to integrate at distinct sites within the human genome. These sites are located primarily in regions that are distant from actively transcribed chromatin and are densely populated with heterochromatin marks. Notably, proviruses in ECs are often integrated into centromeric satellite DNA or specific genes on chromosome 19, which contain zinc finger nucleases (ZNFs) (109, 135). This pattern of integration aligns with the “block and lock” strategy, wherein the proviral genes are silenced by their chromosomal environment, preventing viral expression and replication (136).

PTCs also demonstrate an unusual HIV reservoir profile (137). The size and distribution of the viral reservoir in PTCs are much smaller than those in ART-treated individuals, with some studies showing that the total and intact proviral reservoirs in PTCs are approximately seven times smaller than those in noncontrollers (31). This characteristic is a contributing factor to their ability to maintain HIV control after ART cessation (17, 138). The VISCONTI study reported that PTCs had low levels of HIV DNA that continued to decrease even after the initiation of ART (11). Despite the interruption of ART, PTCs demonstrate a remarkable ability to restrict HIV transcription. And the control mechanisms may occur before treatment interruption (139). These findings suggest that, like ECs, PTCs also achieve viral control through mechanisms of deep latency and transcriptional silencing (49, 140). Research on nonhuman primates also supports the idea that a smaller HIV reservoir is key to achieving post-treatment control of the virus (141). Research on nonhuman primates also supports the idea that a smaller HIV reservoir is key to achieving post-treatment viral control.

Understanding the unique characteristics of HIV reservoirs in ECs and PTCs is critical for the development of new HIV cure strategies. By accurately characterizing the size, distribution, and integrity of these reservoirs, researchers can explore innovative approaches to target and manipulate the reservoirs, potentially leading to a functional cure for HIV. The concept of targeting latent reservoirs through strategies such as “shock and kill” (activating the latent virus and then eliminating it) has been a central focus of HIV cure research. However, the deep latency observed in ECs and PTCs may provide a new avenue for exploring the “block and lock” approach, wherein proviruses are silenced and prevented from being reactivated, thus contributing to long-term viral control. Future studies should focus on improving methods for accurately measuring HIV reservoirs and understanding the mechanisms that contribute to their small size and high integrity in ECs and PTCs. This will allow researchers to identify novel therapeutic targets and refine strategies for achieving sustained remission or even an eventual cure for HIV.

## 5 Defective integrative viruses

Numerous studies indicate that both the host and virus influence disease progression following HIV-1 infection, with attenuated viruses playing an important role (**Table 3**). The number of genome-intact and replication-competent proviruses is significantly reduced in the EC (109, 154). Studies have shown that some LTNPs and ECs have large deletions in the *nef* gene.

**Table 3:** Potential defective integrated virus during viral suppression in HIV controllers.

Sites	Mechanism	References
*nef*	Gene deletion/rare *Nef* polymorphisms downregulate CCR5 and CXCR 4 and increase viral vulnerability to host immunity.	(142)
*vpr*	HIV-1 Vpr upregulates the expression of ligands required to activate the NKG2D receptor and promotes NK cell-mediated killing. The R77Q mutation in the *vpr* gene delays the progression of HIV-1 disease.	(143, 144)
*vif*	HIV-1 *vif* sequences isolated from ECs display relative impairments in their ability to counteract the APOBEC3G host restriction factor compared to *vif* sequences from normal progressors and acutely infected individuals.	(145)
*vpu*	Attenuation of HIV-1 *vpu* alleles; high affinity interactions of KIR; *Vpu* sequence variations impact the downmodulation of HLA-C.	(146–148)
*rev*	Attenuated rev alleles may contribute to viral attenuation and long-term survival of HIV-1 infection.	(149)
*gag*	Gene mutation; CD8^+^ T-cell-mediated escape mutations in *gag* can reduce the HIV-1 replication capacity and alter disease progression. PTCs exhibit more robust Gag-specific CD4^+^ T-cell responses; epitopes of Gag protein-restricted by HLA-B*57 generated a considerable immune response in ECs.	(18, 91, 150, 151)
*po*l	Immune-mediated mutations in *pol* can reduce HIV replicative fitness.	(152)
*env* V1 domain	Long V1 regions play a role in shielding HIV-1 from recognition by V3-directed bNAbs.	(153)

HIV-1 Nef is a small (27–35 kDa) accessory protein that is a crucial auxiliary protein for HIV replication and AIDS development (155). Infection with *nef*-deficient or nef-defective HIV or SIV strains can lead to a slow or nonprogressive disease phenotype (16, 156). This protein is crucial for interacting with the cellular vesicular trafficking system and interfering with cell signaling. Nef engages with proteins related to intracellular trafficking and alters the expression of various cell surface molecules (157). It has diverse *in vitro* functions that influence pathogenesis (158), such as downregulating CD4 (159, 160) and HLA-I (161), upregulating the HLA class II invariant chain (CD74) (162–164), and enhancing viral infectivity and replication (165, 166). The impairment of these Nef activities has been documented in ECs. Compared with those obtained from individuals with chronic progressive infections, Nef clones from ECs presented a markedly reduced capacity to downregulate CD4 (142, 166, 167). Nef facilitates CD4 downregulation by promoting its internalization into the endosome–lysosome compartment, a conserved function that persists during disease progression, thereby increasing viral infectivity and replication.

In general, EC Nef clones are functional; however, Nef clones of HLA-B* 57-expressing ECs may be influenced by host immune selection pressure, resulting in diminished Nef function and a hallmark of the EC phenotype (167). Research has compared chromosomal integration sites and escape mutations between ECs and AIDS patients who need to receive antiretroviral therapy. The team observed differences in HIV integration sites. Intact and defective HIV proviruses from ECs showed a reduced frequency of escape mutations in cytotoxic T-cell epitopes and antibody contact regions. Approximately 15% of intact HIV proviruses in ECs exhibit a *nef* deletion, indicating heightened viral susceptibility to host immune responses due to Nef dysfunction (110).

## 6 Discussion

HIV ECs and PTCs represent important models of sustained HIV remission without ART. ECs represent individuals who are able to naturally suppress HIV replication to undetectable levels without ART. PTCs could represent a more concrete objective for research focused on attaining HIV remission even after ART is stopped. Research on these two groups can provide insights into the immune and genetic factors that enable natural HIV control, which is key for developing therapies that replicate this process in other individuals.

This review outlines the immunological characteristics, genetic variations, and HIV reservoirs associated with HIV ECs and PTCs. Recognizing traits linked to virological control can help identify candidates before ART cessation, whereas targeted mechanistic studies may guide the development of HIV remission therapies. Future studies on immune responses in HIV controllers, for example, could offer valuable insights for discovering new targets for the natural control of HIV-1 infection. ART-free remission is a key goal in HIV research, where patients can maintain undetectable viral loads without the need for ART. ECs and PTCs offer a blueprint for what this could look like. Therapies designed to mimic the immune and genetic traits of ECs and PTCs could offer a path to achieve ART-free remission for a broader population, including harnessing the immune system and genome editing methods for effective virus control.

Although the field of HIV remission without ART has great promise, significant challenges remain, particularly in understanding the complex interplay between genetic, immune, and virological factors. Significant variability in how individuals within these groups respond to HIV is noted, making it difficult to pinpoint universal biomarkers or treatment approaches. Another major limitation in this field is the lack of large, well-characterized cohorts of ECs and PTCs, which hampers the identification of common immunological and genetic traits.

Future research should focus on immune mechanisms, genetic variations, and targeted therapies to eradicate HIV reservoirs, which is crucial in advancing treatment strategies and potentially paves the way for a functional cure. The next steps will involve translating the lessons learned from ECs and PTCs into tangible therapies that could benefit the broader HIV-positive population.
